# Exploring the antifungal activity of the wheat microbiome

**DOI:** 10.1042/BST20250408

**Published:** 2026-09-24

**Authors:** Louise F. Thatcher, Simon R. Law, Lachlan Dow, Lygie Esquirol

**Affiliations:** 1 Commonwealth Scientific and Industrial Research Organisation (CSIRO), Agriculture and Food, Acton, ACT, Australia; 2 Commonwealth Scientific and Industrial Research Organisation (CSIRO), Advanced Engineering Biology Future Science Platform, Acton, ACT, Australia

**Keywords:** hydrolytic enzymes, microbial biological control agent, plant protection, secondary metabolite, volatile organic compound

## Abstract

Wheat (*Triticum aestivum*) is a cornerstone of global food security, yet its productivity is threatened by fungal pathogens such as *Fusarium graminearum*, *Zymoseptoria tritici*, and *Rhizoctonia solani*. These pathogens not only reduce yield but can also compromise grain quality and food safety through the production of mycotoxins. Traditional control strategies, primarily reliant on chemical fungicides and resistant cultivars, face limitations due to evolving pathogen resistance, environmental concerns, and regulatory restrictions. In recent years, the plant microbiome has emerged as a promising frontier in sustainable crop protection. The wheat microbiome, encompassing microbial communities in the rhizosphere, endosphere (root and leaf), and phyllosphere, plays a pivotal role in plant health and resilience. These microbes can suppress fungal pathogens through diverse mechanisms, both directly and indirectly. This mini-review explores the antifungal potential of the wheat microbiome, highlighting key microbial taxa, mechanisms of action with a focus on the underlying biochemistry and molecular processes, and recent advances in omics and microbiome-based biocontrol strategies. This extends to a case study on our own endophyte biocontrol and biochemistry discovery pipeline. We detail the impacts of fungal pathogen exposure and farming practices on wheat microbiome composition and phytopathogen-suppressive traits and highlight pathways for harnessing microbial communities to enhance wheat resistance.

## The importance of harnessing the wheat microbiome

Wheat (*Triticum aestivum*) is one of the most widely cultivated cereal crops globally, providing a major source of calories and protein for billions of people. However, its production is threatened by fungal pathogens such as *Fusarium graminearum* (causing Fusarium head blight), *F. pseudograminearum* (crown rot), *Zymoseptoria tritici* (Septoria tritici blotch), and *Rhizoctonia solani* (root rot and damping-off; Figure 1). These diseases not only reduce yield but can also compromise grain quality and food safety through mycotoxin production, posing significant economic and health risks [1,2].

**Figure 1:**
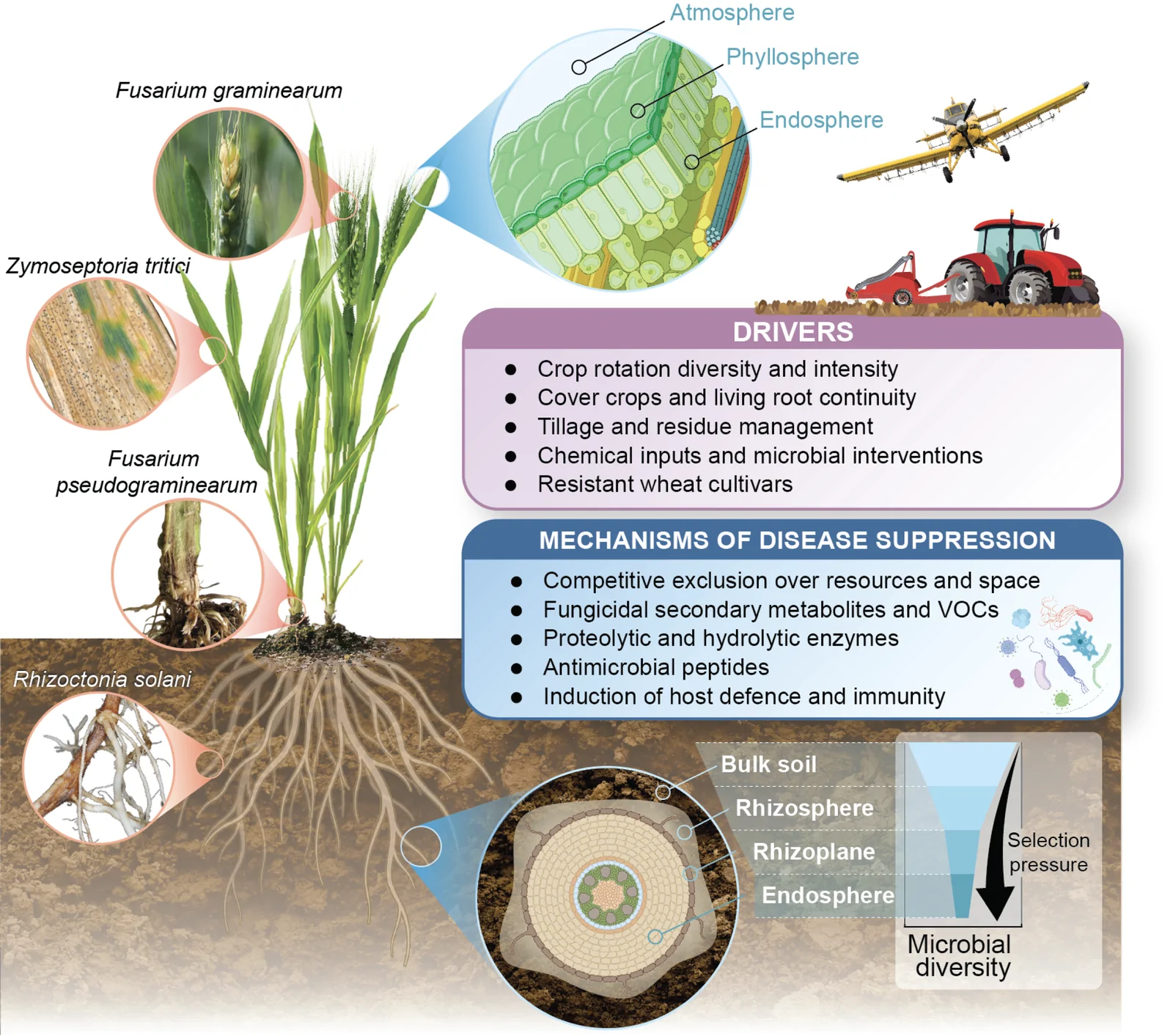
Environment, crop management, and pathogen pressure shape the antifungal potential of the wheat-associated microbiome Wheat plants are exposed to multiple fungal pathogens affecting distinct tissues, including *F. graminearum* (heads), *Z. tritici* (leaves), *F. pseudograminearum* (crowns and stems), and *R. solani* (roots). These infections, together with agronomic drivers such as crop rotation, fallow periods, and targeted synthetic pesticide or microbial interventions, influence the assembly and function of microbial communities across soil–plant compartments (bulk soil, rhizosphere, rhizoplane, and endosphere). Increasing host selection pressure filters microbial diversity from bulk soil toward intimate plant associations, enriching taxa with antifungal functions. The resulting microbiome suppresses pathogen establishment and disease progression through complementary mechanisms, including resource competition, the production of antifungal metabolites and secretion of degradative enzymes, and induction of host defence responses.

Traditional disease management strategies rely heavily on chemical fungicides and resistant cultivars. While effective in the short term, these approaches face critical limitations, including the emergence of fungicide-resistant pathogen strains, environmental and regulatory concerns, and the narrow genetic base of resistance in modern elite wheat varieties [3,4]. These challenges underscore the need for additional crop protection tools or management practices. In this context, the wheat microbiome, comprising diverse communities of bacteria, fungi and other microorganisms that inhabit the rhizosphere, endosphere and phyllosphere, has emerged as a promising frontier for crop protection. Recent advances in high-throughput sequencing and multi-omics approaches have revealed that these microbial communities are not passive occupants but active participants in plant health, capable of shaping disease outcomes and have potential to be harnessed through tailored farm management practices.

## Tools of the trade

The isolation of microbes from wheat tissues or adjacent soils and their subsequent functional characterisation has proven invaluable to understanding their antifungal activities. However, the majority of studies are conducted on single strains and in isolation under *in vitro* conditions, meaning they fail to capture plant interactions and the complex environment in which these microbes naturally exist [5,6]. In many cases, antifungal phenotypes identified in laboratory settings fail to translate when the strain is applied to plants under field conditions [5,7,8]. Perhaps even more confounding is when microbes fail to inhibit pathogen growth in *in vitro* screens but show good efficacy *in planta* [9]. Consequently, our understanding of the antifungal activity of the wheat microbiome, and plant microbiomes generally, has shifted from traditional culture-based microbiology (e.g. isolation of antifungal or disease-suppressive microbes) to culture-independent and *in situ* or *in planta* approaches, including emerging multi-omic approaches, that integrate both microbial and host knowledge to link wheat resistance traits with microbiome-mediated antifungal activity. These include amplicon sequencing, metagenomics, metatranscriptomics, and metametabolomics approaches that have transformed our understanding of the wheat microbiome, revealing not only its taxonomic composition but also its functional potential and active roles in disease suppression. For updates on these technologies, we recommend several recent reviews [10,11].

## Assembly and composition of the wheat microbiome

The assembly and composition of the wheat microbiome is shaped by the interplay of the resident seed microbiome with the soil microbiota, plant host traits, and external conditions, including anthropogenic factors, operating across plant compartments and developmental stages (Figure 1) [12,13]. Soil type and edaphic properties, including moisture, pH, texture, nutrient status, and organic matter, define the primary microbial source pool from which wheat-associated communities are recruited and exert a dominant influence on rhizosphere microbial diversity and composition [12,14].

Amplicon sequencing and metagenomics studies have shown that wheat rhizosphere communities are dominated by Proteobacteria, Actinobacteria, Bacteroidota, and Firmicutes, along with diverse fungi, including Ascomycota, Basidiomycota, and arbuscular mycorrhizal fungi (phylum Glomeromycota), with prokaryotic diversity often 2–3 times greater than that of eukaryotes [15]. Rhizosphere communities are often less diverse than bulk soil, but are more stable and active, with root exudates providing both carbon substrates and signalling molecules that selectively enrich a distinct subset of microbial taxa from bulk soil [16,17]. Within the wheat root endosphere, a further reduction in microbial diversity is observed, but is one more tightly associated with plant function. Endophytic bacteria are frequently dominated by Proteobacteria (e.g. *Pseudomonas*, *Burkholderia*, and *Rhizobium*) and Actinobacteria, while fungal endophytes include non-pathogenic members of Ascomycota such as *Trichoderma* and *Fusarium* [18,19]. Entry into root tissues is imposed by strong host filtering, with evidence from wheat and other cereals suggesting that endosphere communities are more conserved across soils than rhizosphere communities, highlighting host genetic control as an important driver [20–22]. For example, enrichment and depletion of certain microbes in the rhizosphere and endosphere of wheat over 8 years of dryland and irrigation was not a passive process [23], highlighting that plants have the ability to select for certain microbes or that some microbes are better at root colonisation.

In aboveground tissues, the wheat phyllosphere (leaf and stem surfaces) represents a contrasting habitat characterised by low nutrients, larger temperature variation, UV exposure, and desiccation stress, with overall microbial biomass lower than belowground compartments [22,24]. Wheat grains host distinct but comparatively low-diversity microbial assemblages, derived from both systemic colonisation and environmental exposure during flowering and grain fill. These microbiomes are particularly interesting as they can influence mycotoxin risk in grains from phytopathogenic fungi but are also the source of vertical transmission of beneficial microbes to the next generation [25–27].

After harvest, wheat residues, including stubble, become hotspots of microbial activity, supporting diverse saprotrophic bacteria and fungi involved in residue decomposition and nutrient cycling. Community composition changes markedly during residue decomposition and is strongly influenced by environmental conditions such as moisture and temperature, as well as residue-management practices including tillage [28,29]. Importantly, residues can act as both a reservoir for soil-beneficial microbes and fungal pathogens, linking residue microbiomes directly to disease pressure and soil health in subsequent wheat crops [30,31].

## Underlying biochemical and molecular features of an antifungal wheat microbiome

Microbiome-mediated disease suppression can be achieved through multiple mechanisms. Much of what we know has been defined from studies on diverse crops and model plant systems, identifying several shared mechanisms. These include resource competition for nutrients and space, parasitism *via* hydrolytic enzymes, antibiosis *via* secondary metabolites and volatile organic compounds (VOCs), and the induction of defence pathways or systemic resistance in the host plant (Figure 1) [6,32–34]. Microbial communities encompassing Actinobacteria (e.g. *Streptomyces*), Proteobacteria (e.g. *Pseudomonas*), Ascomycota (e.g. *Trichoderma*) and arbuscular mycorrhizal fungi are well known for their abilities to suppress fungal phytopathogens and are well characterised under *in vitro* conditions, with some studied *in planta*. Below we highlight key biochemical and molecular features of these microbes from the wheat microbiome, while we point readers to recent reviews for detailed information on individual biological control methods and potential microbial antagonists of wheat fungal diseases [5,35–38].

The antimicrobial peptide database 6 (https://aps.unmc.edu/) contains over six thousand antimicrobial peptides (AMPs), among which nearly one third are antifungal peptides [39]. These are highly conserved molecules often found across kingdoms. The majority are of animal origin (75%), while only 8% of AMPs in the database are derived from bacteria and 0.5% from fungi, with the remainder from plants (14%) [39]. Of the wheat-associated antifungal proteins identified and studied, the majority are, unsurprisingly, from the plant [40]. However, microbial proteins that are well-studied include enzymes secreted by plant-beneficial microbes when confronted by pathogens. These include proteases that reduce the activity of enzymes produced by pathogens, thereby interfering with infection. These proteases target enzymes, such as exo- and endopolygalacturonase, chitinase, β-1,3-glucanase, and cutinase [8,32,41–43]. Additionally, beneficial microbes produce their own arsenal of hydrolases, for example, those secreted by some *Trichoderma* species as part of their mycoparasitism and destruction of phytopathogen cell walls, leading to hyphal lysis [36,44], as observed microscopically with *Trichoderma* isolates against *Pyrenophora tritici-repentis*, causal agent of tan spot on wheat [45]. Hydrolytic enzymes can also be engineered to provide disease protection, as demonstrated by chitinase overexpression in the wheat colonising endophyte *Phomopsis liquidambaris* leading to improved resistance against *F. graminearum* [46].

Similarly, antifungal small molecules synthesised by microbes within the wheat microbiome can control fungal growth, with bacteria in the genera *Bacillus, Pseudomonas* and *Streptomyces* the most well-studied in this regard. In the rhizosphere, cyclic lipopeptides produced by *Bacillus*, such as surfactin, fengycin, and iturin disrupt the lipid membranes of fungal phytopathogens while also inducing plant resistance [6,8]. *Pseudomonas* species produce various fungicidal small molecules [47], most notably 2,4-diacetylphloroglucinol (DAPG), known to suppress soil-borne plant pathogens in wheat roots [48]. Numerous metabolites of various classes from soilborne *Streptomyces* species inhibit fungal growth, with diverse antifungal polyketide metabolites regularly isolated from rhizospheres [49]. Other metabolites from wheat systems include validamycin (a suppressant of mycotoxin production in *F. graminearum* [50]), or kasugamycin (an inhibitor of pathogen protein synthesis) (reviewed in [5]). Phyllosphere microbes can also produce antifungal metabolites, such as *Pseudomonas piscium*, isolated from wheat heads, which was found to inhibit *Fusarium* growth and mycotoxin biosynthesis via secretion of a phenazine-type antifungal [51]. This compound functions through several mechanisms, including reactive oxygen species generation in target pathogens leading to mitochondrial dysfunction and cell apoptosis [52], as well as targeting histone acetyltransferase resulting in deregulation of histone acetylation and suppression of fungal growth [51]. Recent *in vitro* studies also discovered VOCs that can further inhibit pathogen growth and colonisation, however it remains to be seen if these VOCs are synthesised by microbes *in situ* [6,8,53].

Culture-dependent studies are effective at identifying proteins and small molecules with antifungal activity during *in vitro* cultivation. However, *in vitro* culture conditions do not closely imitate those in field, where biosynthesis responds to biotic [53] and abiotic [54] stimuli, meaning compounds may not be produced under laboratory conditions. As such, culture-independent studies offer an alternative approach to identifying antifungal biochemistry from plant microbiomes by sequencing and assembling biosynthetic gene clusters (BGCs) from entire microbiomes. Recent studies utilising metagenomic and bespoke metabarcoding techniques show that rhizosphere microbiomes contain far larger numbers of BGCs than suggested by *in vitro* experiments, most of which are unknown in terms of their biosynthetic products [55–58]. Very few publications studying the biosynthetic diversity of plant microbiomes focus on wheat, although wheat likely hosts unique microbes with uncharacterised BGCs in similar numbers to other crops [56,57]. It is critical for functional studies to shift from *in vitro* to *in situ* experimental systems to capture the true antifungal diversity of the wheat microbiome, particularly in disease-suppressive soils (discussed in the next section), while a combination of culture-dependent and -independent techniques is required to better characterise secondary metabolism with antifungal potential.

Alternative approaches to understanding antifungal biochemistry from plant microbiomes are emerging, which attempt to circumvent the drawbacks associated with culture-dependent and -independent methods. For example, bipartite cultivation (culturing multiple microbes in a single petri dish or flask, thereby inducing competitive behaviours or antimicrobial gene expression) increases the variety of synthesised bioactive metabolites [59]. More complex synthetic community studies suggest that competition between microbiota can be predicted based on nutrient utilisation, with the prospect of eliminating unwanted taxa [60]. Kemmerling and colleagues [61] explored the correlation of nutrient utilisation and antifungal activity further, using competition assays and nutrient utilisation data. The authors showed that fungi can inhibit wheat pathogens via competition or antifungal activity and that these properties correlated with the nutrient utilisation capability of the inhibitory strain, suggesting that carbon metabolism shapes fungal–fungal interactions.

## Impact of crop management and pathogen exposure

While pathogen exposure generally has negative consequences for the host, including disease development, reduced plant vigour, and yield losses, it can also trigger defensive responses by both the plant and its associated microbiome. As such, pathogen pressure and crop management practices represent converging drivers of microbiome structure and function in wheat systems, collectively shaping disease outcomes and the promotion or degradation of suppressive soils. Continuous wheat cropping, especially when large areas are sown in monoculture of the same cultivar, is associated with the enrichment of soilborne fungal pathogens, with worsening declines in crop yield common after consecutive years of monoculture [62]. However, extended monoculture in wheat can also trigger the development of naturally suppressive soils [13]; the most notable example being take-all decline (TAD), facilitated through the selective enrichment of 2,4-DAPG-producing *Pseudomonas* spp., leading to the gradual suppression of *Gaeumannomyces tritici* [63]. This form of suppression appears to be highly pathogen-specific, as repeated exposure to *R. solani* AG8 and *Fusarium* spp. does not lead to the same agronomically significant levels of TAD, even though it has been shown to reshape the rhizosphere microbiome and enrich antifungal molecules, such as terpenes, non-ribosomal peptides, polyketides, siderophores and post-translationally modified peptides (RiPPS) [64–66]. Although these microbiome shifts may enhance suppressive functions, they do not necessarily offset the disease and yield penalties associated with sustained pathogen pressure. Consequently, crop rotations that increase crop diversity are routinely employed to interrupt pathogen life cycles and promote belowground microbial diversity. Several recent studies have mechanistically linked increased bacterial diversity [67–70] and enrichment of beneficial microbiota that produce antifungal compounds, such as *Bacillus*, *Pseudomonas*, and *Trichoderma* spp [71,72] to the disease-suppressive capacity of soil microbiomes. Studies on reintroduction of wheat-isolated *Trichoderma* or *Streptomyces* biocontrol species to wheat seeds at planting have demonstrated their modulation of endosphere and rhizosphere microbiomes, alongside a reduction in pathogen abundance or disease expression [73,74] and potential to suppress a broader range of wheat pathogens such as *Pythium irregulare*, a soil-borne oomycete pathogen [73]. Interestingly, a study of wheat-maize-soybean rotations coupled with contrasting agricultural management strategies (conventional tillage, no till, low input, and organic farming) found management had a weaker effect than expected, and instead the strongest drivers of microbiome composition were plant organ and plant age [75], highlighting that ecological filtering by the host-plant operates at a finer scale than agronomic practice alone and must be considered alongside rotation design.

Short-term exposure to fungal pathogens also has significant impact on the wheat microbiome. As part of their host infection process, fungal pathogens secrete secondary metabolites or proteins known as effectors that (i) actively kill the plant, (ii) suppress its defence response or alter cell physiology, or (iii) act in a way that shields the pathogen from host detection [76]. Recently, however, it has been demonstrated that some of these effectors attack plant processes whilst simultaneously functioning through antimicrobial activity that affects the host or soil microbiome [67–70]. This is supported by findings that *Z. tritici* infection reshapes the wheat leaf microbiome [77,78], including a significant reduction in community richness, particularly through a depletion of Actinobacteria and Proteobacteria [71]. Wheat fungal pathogens can also harbour mycoviruses that are debilitative to their pathogenicity [79], as demonstrated in an *F. graminearum* strain harbouring a mycovirus, resulting in reduced virulence on wheat coupled with a loss in mycotoxin production [80]. These multi-organism interactions highlight the complexity of plant-microbe interactions but also highlight opportunities to suppress disease in crops.

The growing adoption of no-till and residue retention approaches has been linked to an array of agronomic benefits, including preservation of the vulnerable mycelial networks of arbuscular mycorrhizal fungi and enhanced moisture retention. No-till is also an approach to build soil organic carbon, which is the metabolic reserve that largely underpins microbial biomass, biodiversity, and ecosystem services such as disease suppression [81]. For example, long-term adoption of no-till and residue retention approaches was shown to build the disease suppressive capacity of low-fertility soils in low rainfall regions (long thought unlikely to support suppressive microbial communities) to agronomically effective levels against Rhizoctonia bare patch in wheat [82]. However, the relationship between conservation practices and antifungal activity is not uniformly positive, as wheat residues can also serve as a ‘brown bridge’ that enables the persistence of pathogens between cropping generations. This necessitates strategies to accelerate residue degradation and thus reduce primary inoculum, such as strategic tillage, which when used sparingly, can temper soilborne disease pressure without fully eroding the microbiome-mediated benefits of no-till [83]. Additionally, there is growing interest in biocatalysts, e.g. laccase-based products, and synthetic communities (SynComs) of living microbes with enhanced cellulolytic and ligninolytic capacity to accelerate wheat residue decomposition.

Pesticide application, including fungicides, often leads to reduced soil and rhizosphere microbial abundance and diversity (amongst other organisms), altered community composition, and decreased microbiome network complexity [77,84–86]. They can impact non-target and plant-beneficial microbes either directly or indirectly through interference with interspecies interactions, such as communication and metabolic cooperation, where metabolites or nutrients produced by one species are used as substrates by a neighbouring species [87,88]. Understanding the impacts of fungicide use on other interventions, such as biocontrol, is critical for designing integrated disease management strategies that balance chemical control with microbiome conservation.

External factors can also impact the wheat microbiome indirectly. Wheat actively influences microbiome assembly through the release of root exudates, which serve as nutrient sources and signalling molecules for rhizosphere microbes. Exudates include both primary and secondary (or specialised) metabolites, and their composition varies with plant developmental stage, genotype, nutrient availability, soil physicochemical properties, as well as exposure to biotic and abiotic stresses, leading to shifts in microbial recruitment and activity [89,90]. This highly dynamic nature of wheat root exudates is a key mechanism through which the host influences ecological filtering. Supporting this, Regmi et al. [91] demonstrated that wheat-derived exudates can restructure rhizosphere microbial communities and modulate pathogen abundance in a soil-dependent manner, underscoring the reciprocal relationship between host physiology and microbiome assembly. Consequently, the spatial and temporal dynamics of the wheat microbiome likely reflect not only environmental filtering but also environmentally mediated changes in host exudation patterns.

## Harnessing the wheat microbiome into biochemical solutions—an endophyte case study

We detail here a pipeline, based on our own work, for discovery and characterisation of the antifungal biochemical potential within the wheat microbiome (Figure 2). **Stage 1** started with site and sample selection. We employed a series of targeted approaches to select for and curate a collection of high-performing microbial isolates associated with long-term wheat health. Firstly, crop fields that showed consistent high performance in crop health and grain yield were selected, from which non-diseased wheat plants were harvested. Microbe isolation targeted endophytic microbes from the wheat roots on the premise that they provide a benefit to the plant and do not harm it; specifically, we selectively isolated microbes from the phylum Actinobacteria, known for plant beneficial traits and production of antifungal compounds [5,92].

**Figure 2:**
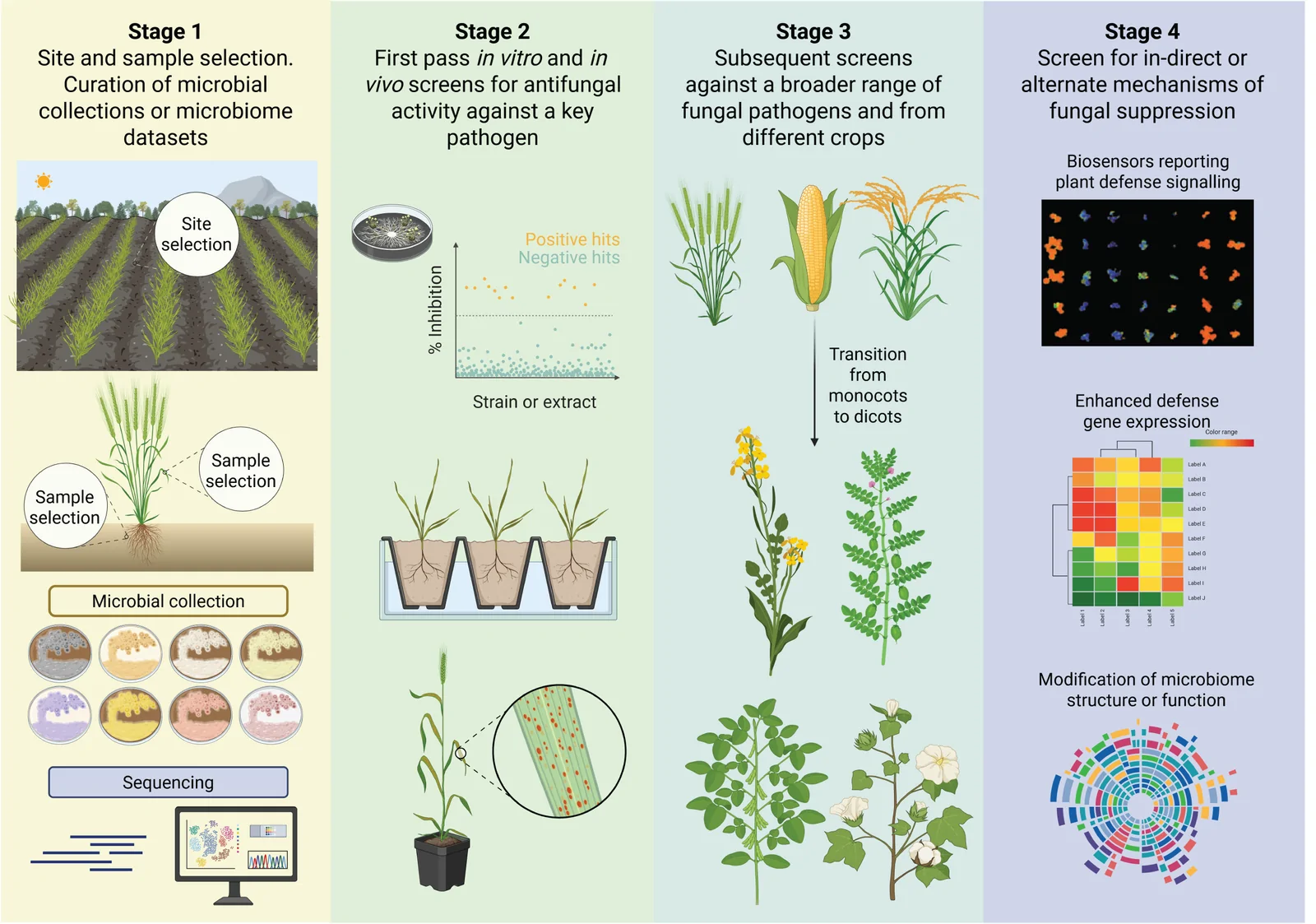
Pipeline for discovery and characterisation of antifungal biochemical potential within the wheat microbiome Highlighted is a staged approach, from targeted data or microbial sample collection (Stage 1) to first pass antifungal screens against a strategic wheat pathogen to identify leads (Stage 2). Subsequent stages are characterised by broadening antifungal assays towards suppression of other wheat fungal pathogens (from biotrophs such as rusts to necrotrophs such as *Fusaria* species) and a transition to pathogens of other monocots (e.g. corn, rice) as well as dicot crops (e.g. oilseeds, legumes, and fibre crops) (Stage 3). Characterisation of more complex wheat microbiome antifungal traits can be explored in Stage 4 by investigating if and how a microbial strain modifies plant defence signalling or gene expression, or how it may modify the overall microbial structure or behaviour of the wheat microbiome towards antifungal traits.

**Stage 2** involved parallel *in vitro* and *in vivo* antifungal screening of the collection, initially focussed on one economically important pathogen: the wheat crown rot pathogen *F. pseudograminearum*. Screens identified two *Streptomyces* sp. strains (MH71 and MH243) with strong antifungal activity in a laboratory setting (>95% inhibition of mycelial growth) that translated to a 25% reduction in disease severity on wheat plants [92]. Subsequent multi-omic studies attributed MH71 antifungal activity in part to the VOC tropone and several secondary metabolites, including the polyketides nigericin and several niphimycins, but genome sequencing suggests much broader metabolic potential with MH71 encoding 50 predicted BGCs [93].

**Stage 3** expanded antifungal screens beyond *F. pseudograminearum*, identifying strains capable of producing a suite of antifungal compounds that suppress a broader range of fungal pathogens beyond those that infect wheat or other cereals, including those that cause root or foliar disease in canola, chickpea, or cotton [94–98]. Focusing on the antifungal compounds secreted by Actinobacteria, we found that the filtered fermentation extracts of *Streptomyces* sp. strains MH71 and MH243 were highly effective in suppressing *Sclerotinia* stem rot disease on canola plants, highlighting the broad antifungal abilities of some wheat microbiome members [94].

To assess the indirect ability of isolates in our collection to suppress fungal pathogens, in **Stage 4** we screened a subset of strains *in planta* for activation of plant defence signalling and ability to stimulate plant immunity. This identified that pretreatment of plants with several strains, including *Streptomyces griseoaurantiacus* strain MH191, prior to fungal pathogen exposure led to enhanced plant defence gene expression. Meanwhile, *S. griseoaurantiacus* concomitantly produced fungicidal and fungistatic metabolites such as undecylprodigiosin, and manumycin-related compounds, along with antifungal VOCs such as dimethyldisulfide and the unusual 2,4,6­trimethylpyridine [95,97,98].

This case study highlights the wheat microbiome as a rich, underexplored source of antifungal compounds and immune-modulating metabolites with broad applicability in cropping systems, and shows that progressing this work depends on multidisciplinary science approaches and a wholistic research strategy (Figure 2).

## Summary and conclusions

The wheat microbiome is a critical yet underutilised component of disease control in one of the world’s most important staple crops. As the efficacy of traditional synthetic fungicides continues to erode, microbiome-mediated suppression of fungal pathogens offers a complementary and potentially durable route to safeguarding wheat productivity while reducing chemical inputs. Current evidence indicates that antifungal activity within the wheat microbiome arises from the combined action of ecological and biochemical processes. Functions may be spatially and temporally structured across plant compartments, with the presence and abundance of microbes associated with these functions dynamically reshaped by pathogen pressure, crop management practices and environmental context, underscoring the need to move beyond single-strain paradigms towards community- and mechanism-informed frameworks. This includes building disease-suppressive soils, microbiome-compatible biocontrol agents (either single or consortia), or direct deployment of microbial-derived chemistries, which, integrated with informed crop management practices, represent a promising pathway towards resilient and sustainable wheat production.

## Perspectives

- The wheat microbiome encompasses diverse microbial communities that play a pivotal role in the suppression of economically destructive fungal diseases whilst counteracting the loss in efficacy of traditional crop protection strategies reliant on chemical fungicides.
- During this review we note studies reporting key microbial taxa linked to wheat microbiome antifungal activity, yet a disparate knowledge gap in how these microbes function *in situ* at the biochemical or molecular level. This gap highlights potentially underutilised tools for wheat disease control alongside the opportunity to integrate knowledge from other important monocot crops such as barley (*Hordeum vulgare*), rice (*Oryza sativa*), and maize (*Zea mays*), as well as from model grass species such as *Brachypodium distachyon*.
- Multi-omics technologies have significantly advanced our ability to study the antifungal activity of the wheat microbiome, particularly from unculturable microbes. Research should now shift to how we integrate the recruitment of beneficial microbiome traits alongside conventional disease resistance breeding in wheat, followed by crop management practices synergistic to microbiome-mediated or microbiome-derived (e.g. biochemical) suppression of wheat fungal pathogens.

## Open Access

Open access for this article is provided via our Subscribe to Open programme, made possible through the support of our subscribing institutions. Learn more here: https://portlandpress.com/pages/open_access_options_and_prices#conditional.

## Abbreviations

AMPs: antimicrobial peptides
BGCs: biosynthetic gene clusters
DAPG: 2,4-diacetylphloroglucinol
TAD: take-all decline
VOCs: volatile organic compounds
